# Predicting failure loads of graphene incorporated adhesively bonded single lap joints fabricated with short glass fibre reinforced polylactic acid using ANN approach

**DOI:** 10.1038/s41598-025-02246-x

**Published:** 2025-12-31

**Authors:** Thulasidhas Dhilipkumar, Abdellatif M. Sadeq, Arun Prasad Murali, Karthik V. Shankar, P. Karuppusamy, Murugan Rajesh, Mohammad Rezaul Karim, Karuppaiah Selvakumar, N. Dinesh kumar

**Affiliations:** 1 Department of Mechanical Engineering, Amrita Vishwa Vidyapeetham, Amritapuri, Kollam, 690525 Kerala India; 2 Centre for Flexible Electronics and Advanced Materials, Amrita Vishwa Vidyapeetham, Amritapuri, Kollam, 690525 Kerala India; 3 Mechanical and Industrial Engineering Department, College of Engineering, Qatar University, Doha, Qatar; 4 Department of Mechanical Engineering, Vel Tech Rangarajan Dr. Sagunthala R&D Institute of Science and Technology, Chennai, 600062 Tamilnadu India; 5 Department of Chemistry, Centre for Research and Development, Vinayaka Mission’s Kirupananda Variyar Engineering College, Vinayaka Mission’s Research Foundation (DU), Salem, 636308 Tamil Nadu India; 6 School of Mechanical Engineering, Vellore Institute of Technology, Vellore, 632014 Tamilnadu India; 7 Center of Excellence for Research in Engineering Materials (CEREM), Deanship of Scientific Research, King Saud University, 11421 Riyadh, Saudi Arabia; 8 Department of Physiology, Saveetha Dental College and Hospitals, Saveetha Institute of Medical and Technical Science (SIMATS), Saveetha University, Chennai, 600077 India; 9 Department of Chemistry, Nanomaterials Laboratory, Kalasalingam Academy of Research, and Education, Krishnankoil, Srivilliputhur, 626126 India

**Keywords:** Additive manufacturing, Graphene, Adhesive bonding, Shear test, ANN, Mechanical engineering, Structural materials, Engineering, Materials science

## Abstract

Additive manufacturing has been prominent for making complicated polymeric structures, with PLA being preferred for its biodegradability, ease of use, and wide 3D printing compatibility. The present study aims to explore the effects of graphene-integrated adhesive on shear properties, failure modes, and vibrational response of adhesively joined bonded joints prepared with 3D printed short glass fibre-reinforced polylactic acid (PLA) adherents. Field emission scanning electron microscopy (FESEM) was used to analyse fracture surfaces, while artificial neural networks (ANN) predicted failure modes using a backpropagation algorithm. Tensile testing of bulk samples indicated that samples printed with 0º raster orientation have higher tensile strength (30.7 MPa) than samples printed with 45º (26.7 MPa) and 90º (23.4 MPa) raster orientations. Shear test results demonstrate that incorporating 1.0 wt.% of graphene into the adhesive enhances the adhesive joint’s shear properties, leading to a 19.51% increase in shear strength compared to neat samples. The free vibrational analysis avowed that the addition of graphene up to 1.0 wt.% increases natural frequencies due to improved stiffness from its well-dispersed state within the epoxy matrix. Furthermore, the failure load was accurately predicted using an artificial neural network trained on data from stress–strain curves. The R^2^ value of 0.9861 indicates that the results are reliable and show a good correlation. Thereby, this study demonstrated how graphene-integrated epoxy adhesives enhance the mechanical and vibrational properties of adhesively bonded lap joints prepared with 3D printed short glass fibre-reinforced PLA adherents, while also using artificial neural networks to predict failure modes, providing a novel approach to optimise the performance of adhesively joined 3D printed components.

## Introduction

The emergence of 3D printing, particularly Fused Deposition Modeling (FDM), has transformed manufacturing by providing a more direct and efficient method for creating components. Unlike traditional methods requiring specialised tooling and fixtures, 3D printing allows the production of components directly from CAD designs^1–3^. This versatility enables rapid prototyping, customisation, and the development of complex geometries that would be difficult to achieve using conventional techniques. Thermoplastic filaments, such as Polycarbonate (PC), PLA, Polyamide (PA), and Acrylonitrile Butadiene Styrene (ABS), are frequently utilised in FDM processes^4,5^. However, the overall performance of thermoplastic components produced by FDM is often below standard, which restricts their potential applications. To enhance the mechanical properties of pure thermoplastic parts manufactured by FDM, the filament feedstock is typically strengthened with different fibre materials, including nanofibers, short fibres, and continuous fibres^6,7^. Among these choices, 3D-printed short fibre-reinforced thermoplastic composites, particularly prepared with PLA, are gaining popularity for structural load-carrying applications^8^. PLA offers a unique combination of biodegradability, ease of manufacturing, and strong mechanical properties, which can be enhanced through fibre reinforcement. This makes PLA-based composites a viable choice for developing lightweight, eco-friendly, and high-performance structural components^9^. However, the bed size limitation of printers is a major limitation that limits their usage in many weight-sensitive engineering applications^10,11^. To address the size limitations of FDM printers, adhesive bonding enables the assembly of multiple components into larger structures^12,13^**.** This approach is particularly valuable for creating large-scale parts that would be impractical to produce in a single piece^14^.

Adhesive bonding is a critical part of 3D printing, especially for assembling components into larger structures. It offers superior properties such as enhanced fatigue resistance and damage tolerance compared to traditional joining methods, making it suitable for applications where reliability and durability are essential^15,16^. By combining 3D printing with adhesive bonding, manufacturers can produce complex and high-performance parts tailored to specific needs. This approach, which leverages the versatility of 3D printing to create custom components with intricate designs, is driving innovation in various industries, including aerospace, automotive, medical, and construction^17–19^.

The most commonly used joint configuration is the single lap joint (SLJ) due to its ease of manufacturing and functionality^20^. Over the decades, improving adhesive-bonded SLJ strength and durability has been a critical concern^21^. It has been a common practice that adherents and adhesives are graded in terms of their composition and geometry to improve the structural integrity of joints^22,23^. Many researchers have tried to improve adhesive joint performance by altering the geometry of the adherends, adding filler materials, incorporating beads, adding fillets to the ends of the adhesive that overlap, or altering the joint structure^24–33^. For instance, Benli et al.^34^ tested PLA-based filaments containing various fillers and infill ratios to make adhesive joints at different temperatures. Their findings showed that the strongest bond, measuring 4.14 MPa, was achieved with carbon fibre (CF) adherents at a 100% infill ratio at room temperature. On the other hand, the weakest bond, measuring 2.05 MPa, was observed with PLA plus (PP) adherents at a 50% infill ratio at −25 °C.

Tiwary et al.^35^ examined the adhesive bonding of dissimilar 3D-printed materials (ABS, PLA) using various joint designs, adhesives, and surface treatments. Results indicated that ABS with a stepped joint, plasma-treated, and bonded with Loctite adhesives provided the best performance. Kajimoto et al.^36^ reported that strength loss can be minimised by increasing the length of the lap joint and incorporating an extra layer prior to the epoxy resin being cured. Kemiklioğlu et al.^37^ reported that auxetic adherends demonstrate superior strength compared to flat surfaces. Notably, the strength of ductile adhesives in auxetic bonded joints increases with adhesive thickness, while the strength of brittle adhesives decreases with thicker auxetic bonds. Özkan Öz et al.^38^ revealed that increasing the adherent thickness, the overlapping area, and the raster angle improved the strength of joints. Özkan Öz et al.^39^ analysed the mechanical and joint characteristics of 3D-printed PLA combined with carbon fibre powder (CFP). Adding CFP significantly improved the properties of the composite. Furthermore, the presence of polyethylene oxide enhances flexibility, although it leads to a decrease in strength. Naat et al.^40^ studied bio-inspired surface textures from fish scales and tree frogs on 3D-printed polycarbonate specimens. The results showed that these textures improved shear strength by 242% and 283% compared to polished surfaces. Khosravani et al.^41^ found that making steps in the assembly region significantly impacts the fracture load of 3D-printed adhesive joints.

Previous studies have explored the performance of adhesively bonded joints in 3D-printed assemblies^42–44^. However, limited research has focused specifically on the synergistic effects of using nanofiller-reinforced adhesives in combination with short fibre-reinforced 3D-printed PLA adherents. Furthermore, only a few researchers have investigated the development of high-performance prediction models using advanced deep-learning techniques based on ANN to assess the performance of adhesively bonded joints under shear loading^26,45^. ANN is an excellent approach for analysing the complexities of such predictions. This method is particularly valuable for addressing nonlinear problems using nonlinear regression techniques currently applied in various engineering fields.

The present study addresses this research gap by investigating the impact of graphene nanopowder as a reinforcing agent in the epoxy adhesive used to bond single-lap joints composed of short glass fibre-reinforced PLA adherents fabricated using fused deposition modelling (FDM). The current research aims to evaluate the influence of graphene on shear strength, failure modes, and vibrational characteristics of adhesively joined lap joints prepared with 3D printed short glass fibre-reinforced PLA adherents. Graphene was incorporated into the epoxy adhesive at various weight fractions (0.5 wt.%, 1.0 wt.%, 1.5 wt.%, and 2.0 wt.%) to assess its effect on joint performance. Field emission scanning electron microscopy (FESEM) was used to characterise fracture surfaces and crack propagation behaviour. Modal analysis was conducted to determine changes in vibrational response due to modifications in joint stiffness. Furthermore, failure load in adhesively bonded lap joints made from 3D-printed short glass fibre-reinforced PLA adherents was predicted using ANN and backpropagation techniques.

## Materials and methods

The short glass fibre-reinforced PLA was provided by eSUN, China. The ePLA filament contains 16% glass fibre by weight. The properties of PLA-Glass fiber filament are given in Table 1. The graphene powder, with a purity exceeding 99%, a surface area of 80 m^2^/g, and a density of 0.25 g/cc, was sourced from BT Corp, Bengaluru, India. The two-component epoxy adhesive (LY556) with hardener (HY951) was procured from SM Composites, Chennai, India. The properties of the adhesive showed in Table 2.

**Table 1:** Properties of PLA-Glass fibre filament (As per manufacturer’s data sheet).

Sl No	Density (g/cm^3^)	Tensile strength (MPa)	Elongation at break (%)	Flexural strength (MPa)	Flexural modulus (MPa)	Heat distortion temperature (℃)
1	1.31	59.27	7.99	85.01	4414.89	55

**Table 2:** Physio-mechanical properties of epoxy adhesive^46,47^**.**

Property	Value	Unit
Flexural Strength	110–120	MPa
Interlaminar shear strength	62–66	MPa
Density	1.15–1.2 at 25 °C	g/cm^3^
Curing time	24	Hours
Viscosity	10,000–12,000	mPa s
Flash point	110 °C	°C
Glass Transition Temperature (Tg)	175–185	°C

### Adhesive preparation

**Fig. 1:**
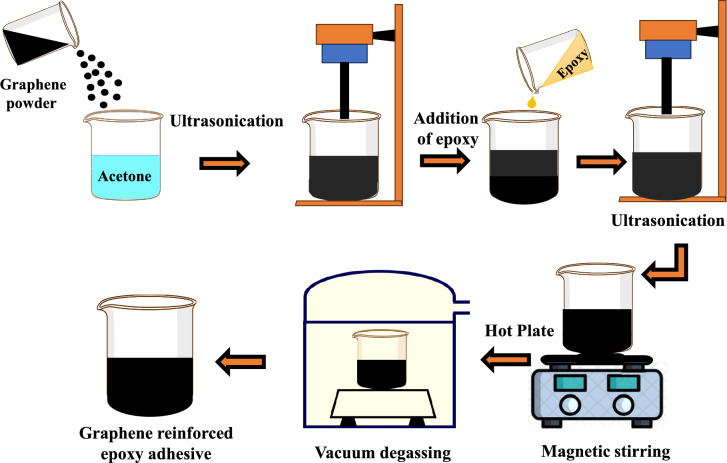
Graphene/epoxy adhesive preparation process.

The graphene was dispersed in the five different weight concentrations (0%, 0.5%, 1.0%, 1.5% & 2.0%) with epoxy adhesive using ultrasonication. Initially, graphene was mixed with high-purity acetone and sonicated at 40 Hz for 60 min, with sonication paused every 10 min to manually stir the mixture using a glass rod to enhance dispersion. Then, the required amount of epoxy was added to the sonicated mixture, and the process was repeated for another 60 min. The sonicated mixture was then placed on a hot plate magnetic stirrer to ensure the uniform dispersion of graphene nanopowder in the epoxy. Finally, the sonicated mixture was placed in a vacuum-degassing oven to remove any entrapped air. The graphene/epoxy adhesive preparation is shown in Fig. 1.

### Sample preparation

The 3D printed samples were made using the MD-400D IDEX Hyper Speed 3D Printer from China. First, the CAD models of the specimens were created using SolidWorks 2022. Then, the part design was sliced using Ultimaker Cura 5.7.2 software to set printing parameters. The printing parameters used to prepare the specimens are listed in Table 3**.**

**Table 3:** FDM Printing Parameters for fabricating bulk specimens^13^.

Sl No	Printing parameters	Value
1	Infill %	100
2	Print direction	[0°, 45°, 90°]
3	Infill rate	60 mm s^−1^
4	Outer shell speed	30 mm s^−1^
5	Nozzle diameter	0.4 mm
6	Extruder temperature	220 ºC
7	Bed temperature	60 ºC
8	Layer thickness	0.1 mm

**Fig. 2:**
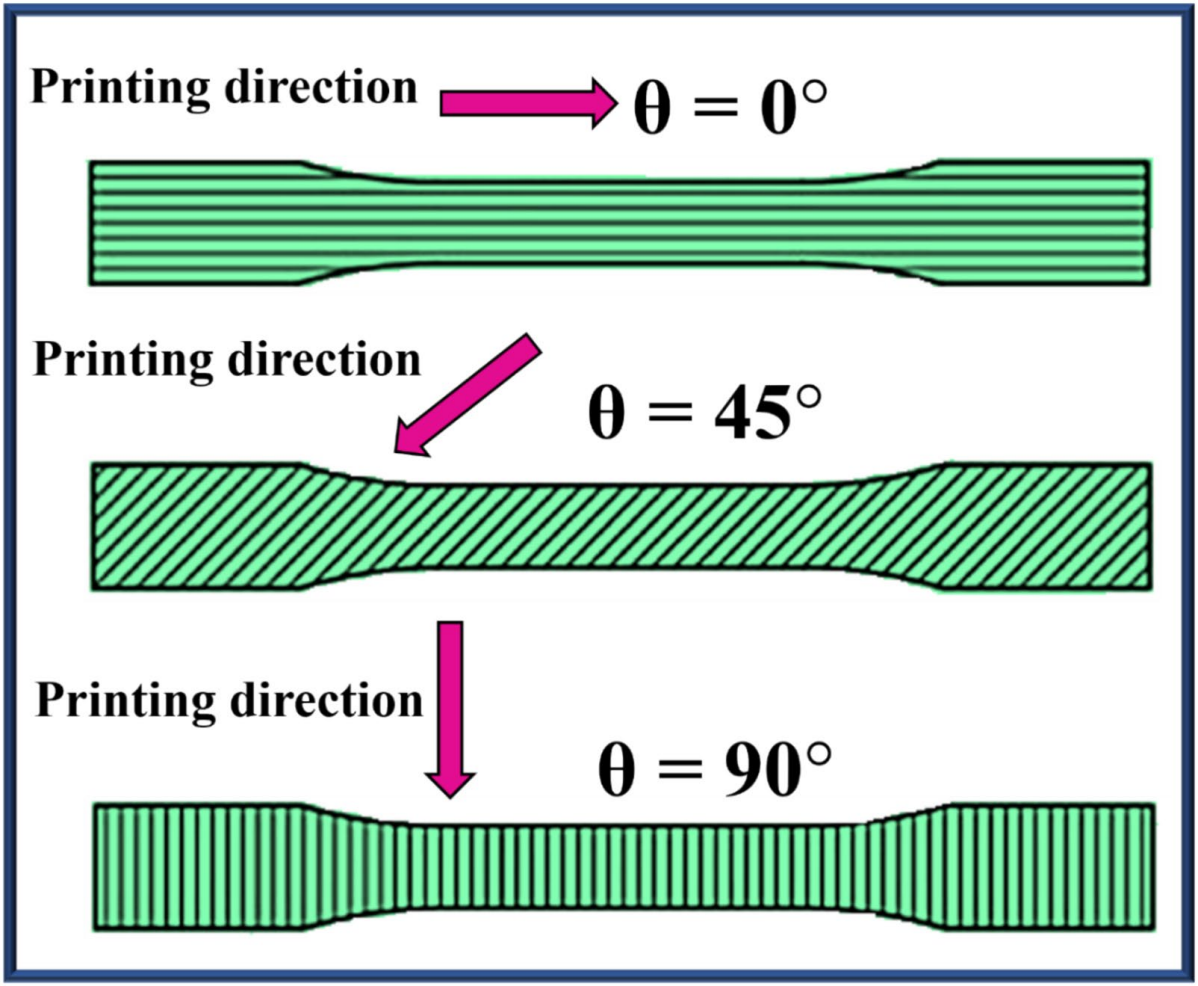
Printing orientation of bulk samples.

To investigate the impact of printing parameters on the tensile properties of short glass fibre-reinforced 3D-printed PLA, bulk samples were initially prepared in 0º, 45º, and 90º raster orientations as shown in Fig. 2. The 0º raster orientation was selected based on present and previous experimental results^48^ to prepare the adherents for a single-lap joint using the FDM technique.

**Fig. 3:**
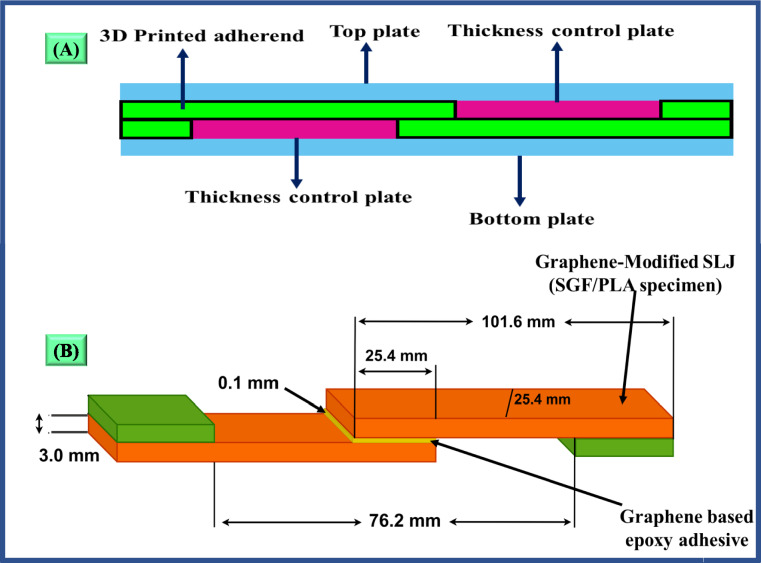
Fabrication of lap joints (**a**) Steel plate arrangement (**b**) Configuration of shear test specimen.

Before joining, the 3D-printed short glass fibre-reinforced PLA adherents were abraded using a P400 abrasive sheet to enhance the wettability of the overlapping area. The abraded area was then cleaned using high-purity acetone and wiped with a cotton cloth. A neat and graphene-mixed adhesive was applied to the overlapping area of 3D-printed adherents using a brush. After that, specially designed steel plates were used to set adherents in a lap joint manner (Fig. 3a), and 0.1 mm glass beads were used to control the adhesive thickness. After 4 h of pre-curing at 50 °C in an industrial oven, the pre-cured lap joints were allowed for 24-h post-curing at atmospheric temperature. The configuration of single lap joint specimens is shown in Fig. 3b.

### Mechanical testing

The 3D-printed bulk and adhesively bonded single-lap joint specimens were tested using a Tinius Olsen universal testing machine with load cells of 10 kN and 50 kN, respectively. The samples were tested with a displacement rate of 1 mm/min. The testing was performed in accordance with ASTM D 638 and ASTM D 5868 standards. Each sample underwent testing three times, and the average values were reported in the study.

### Free vibrational analysis

**Fig. 4:**
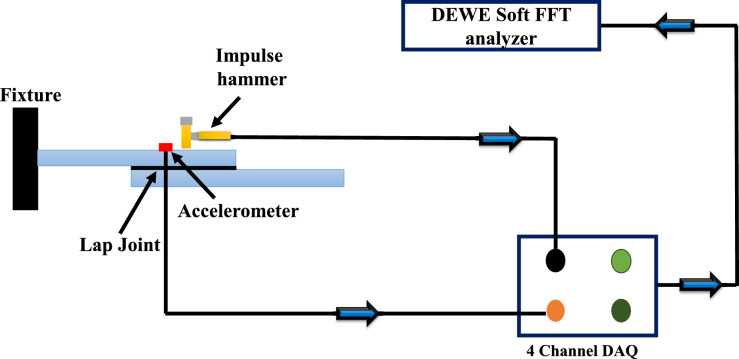
Experimental modal analysis setup.

To investigate the vibrational behaviour of adhesively bonded lap joints made from 3D-printed short glass fibre-reinforced PLA adherents, modal analysis was performed under fixed–fixed boundary conditions. Initially, the samples were divided into 10 nodal points, and an impulse hammer was used to excite the specimens at these nodal points. Special wax was used to place the accelerometer sensor on the specimens. A four-channel data acquisition system was used to capture the displacement signals, which were then converted into frequency response functions using the FFT algorithm. Dewesoft X was used to calculate the natural frequency, mode shapes, and damping values of adhesively bonded lap joints made from 3D-printed short glass fibre-reinforced PLA adherents. The experimental setup of modal analysis is illustrated in Fig. 4.

### Microstructural analysis

The fracture surfaces were analysed using FE-SEM to determine the micro-mechanisms. To enhance conductivity, the samples were coated with chromium through sputter coating. Each sample was prepared with dimensions of 10 mm × 10 mm × 2.5 mm.

### Artificial neural networks

**Fig. 5:**
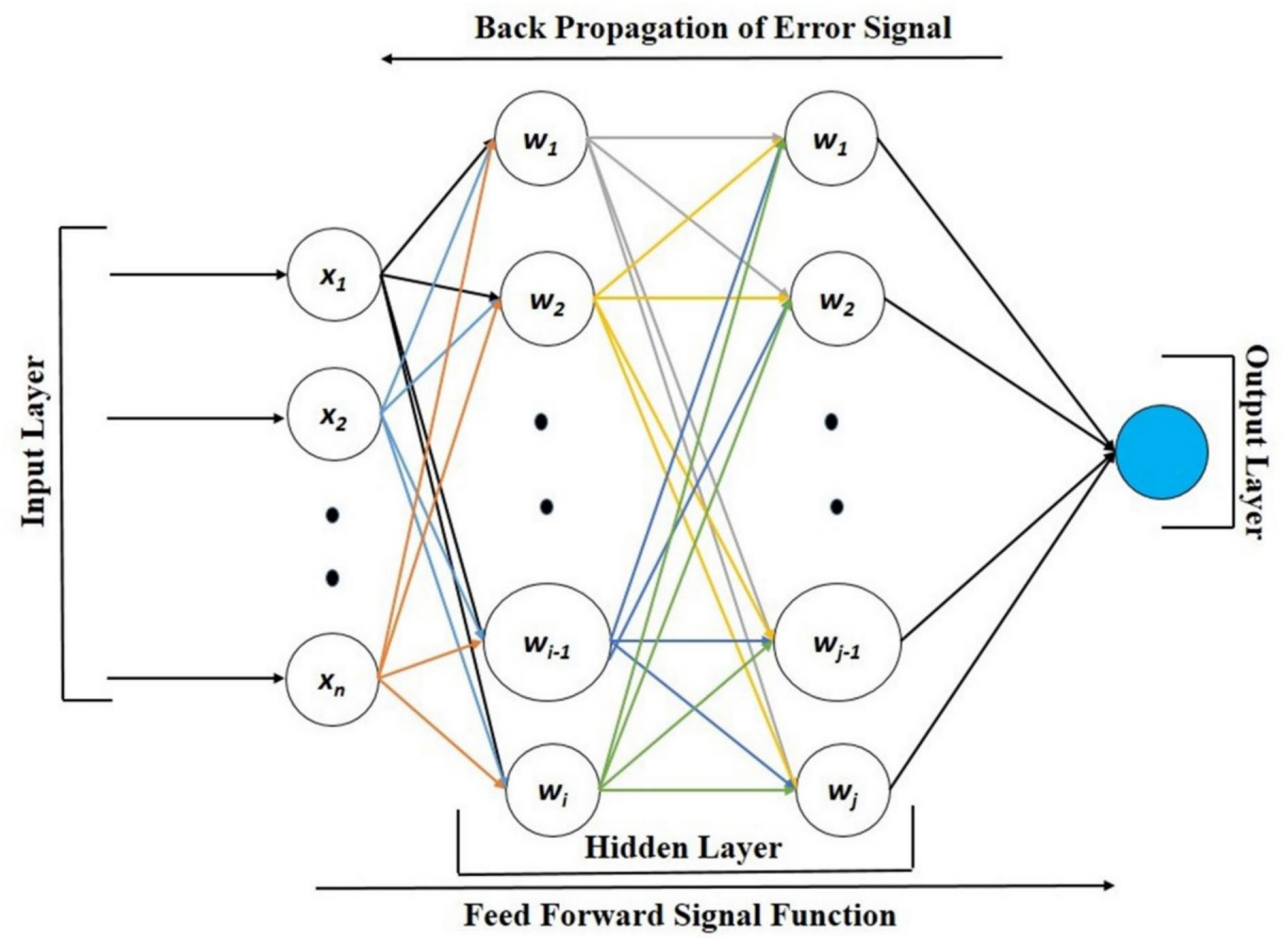
Schematic representation of artificial neural networks.

The current investigation used Python programming to construct an Artificial Neural Network (ANN) using a Jupyter notebook environment. The error signal generated within the ANN is distributed using the backpropagation technique, which facilitates the adjustment of weights and biases. The implementation utilises NumPy to handle matrix computations. The input layer consists of five different combinations of graphene-incorporated adhesively bonded single-lap joints. Meanwhile, the network includes 64 hidden layers and an output layer that represents displacement, as shown in Fig. 5. The process is executed using an optimisation method based on gradient descent. The ANN model is trained on a total of 25 datasets, with 70% allocated for training, 15% for validation, and the remaining 15% for testing.

## Results and discussions

### Bulk specimen test

The tensile properties of short glass fibre-reinforced PLA samples are significantly influenced by the printing orientation, which affects the alignment of fibres and polymer layers within each 3D-printed structure. In the 0º orientation, the fibres are aligned parallel to the direction of the applied load, allowing them to carry maximum load. The glass fibres are stiffer and stronger than PLA. Thus, they serve as the primary load-bearing member in this orientation. This alignment aids in efficient load transfer along the fibre axis, enhancing tensile strength and energy absorption. Consequently, samples printed at 0º exhibit a greater load-carrying capacity and superior resistance to tensile loading.

Conversely, when samples are printed in a 90º orientation, the fibres are positioned perpendicular to the applied tensile force, which limits their ability to contribute to load-carrying. Because of this reason, the PLA matrix absorbs more tensile load, which impacts mechanical performance, as PLA does not possess the tensile strength of glass fibres. This inefficiency in load transfer leads to reduced tensile strength and lower energy absorption in 90º-oriented samples. As a result, these samples tend to fail at lower stress levels and exhibit reduced strain values compared to those printed at 0º and 45º orientations. Thus, it signifies the importance of fibre orientation in load transfer effectiveness within composite materials.

The quality of interlayer bonding also plays a crucial role in determining tensile strength, particularly in 3D-printed samples where bonding between layers can vary based on orientation. In the 0º orientation, the continuous strands and aligned fibres along the length of the sample lessen the reliance on interlayer bonding strength to withstand tensile loads. Here, stress is distributed more uniformly along the fibre direction, thereby improving overall mechanical performance. However, in samples printed at 90º, the tensile load depends on the bonding between layers, as the primary load-carrying fibres lie perpendicular to the applied force. This orientation creates limited bonding areas in the loading direction, leading to stress concentration points that increase the risk of fracture. Due to this reason, the samples printed at 90º exhibit lower tensile strength and strain.

**Fig. 6:**
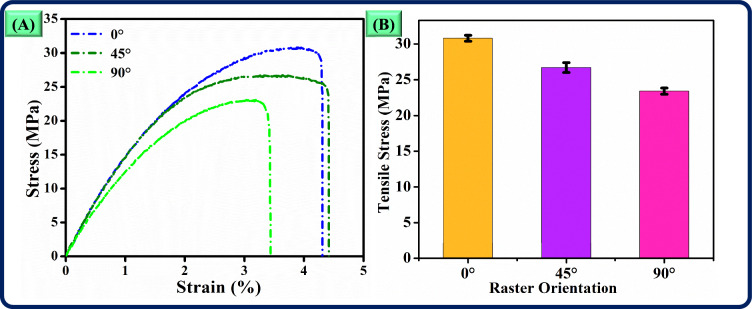
Tensile properties of short glass fibre-reinforced PLA samples (**a**) stress vs strain, (**b**) tensile stress vs raster orientation.

The differences in energy absorption are closely related to the fracture behaviour of each orientation. Energy absorption capacity depends on the material’s ability to deform before failure. For the 0º samples, the continuous fibre alignment provides resistance to crack propagation, enabling the material to endure higher levels of deformation and absorb more energy, as indicated by the larger area under the load versus displacement curve. In contrast, 90º oriented samples lack this resistance, allowing cracks to propagate easily through the weaker interlayer bonds. Thus, it results in lower energy absorption and premature fracture, alongside reduced strain at break, as shown in Fig. 6a.

Moreover, the tensile strength data further underscores the impact of printing orientation on mechanical properties (Fig. 0.6b). With a tensile strength of 30.7 MPa, the 0º oriented samples demonstrate a 14.98% improvement over the 26.7 MPa observed in 45º samples and a 31.19% increase over the 23.4 MPa strength in 90º samples. This increase emphasises the reinforcement potential of glass fibres when optimally aligned with the loading direction, as in the 0º orientation. Meanwhile, the 45º samples show intermediate performance, as their fibres are positioned at an angle to the tensile load, allowing some degree of load resistance but not as effectively as the fully aligned 0º samples.

In summary, the variations in tensile properties across different printing orientations (0º, 45º, 90º) can be attributed to fibre alignment relative to the applied load, interlayer bonding strength, stress distribution, and energy absorption characteristics. These findings emphasise the critical role of printing parameters in optimising mechanical properties, especially for composite materials like short glass fibre-reinforced PLA.

### Shear analysis

**Fig. 7:**
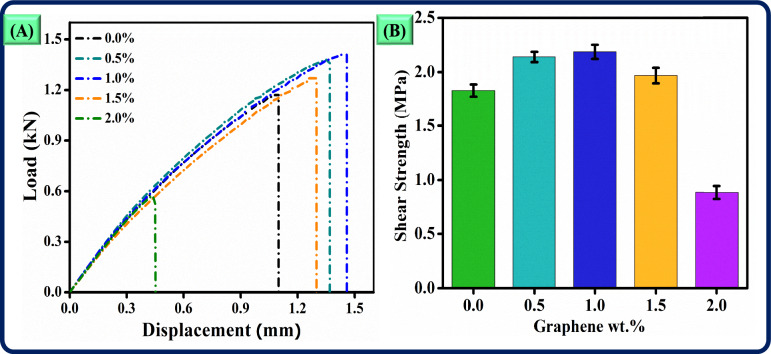
Shear properties of adhesively bonded lap joints made from 3D-printed short glass fibre-reinforced PLA adherents (**a**) load vs displacement, (**b**) shear strength vs graphene weight concentration.

The addition of graphene has significantly influenced the shear behaviour of adhesively bonded lap joints made from 3D-printed short glass fibre-reinforced PLA adherents, impacting the load-carrying capacity, strain values, and energy absorption (Fig. 7a). Results demonstrate that incorporating a 1.0 wt.% concentration of graphene into the adhesive enhances the joint’s shear properties, leading to a 19.51% increase in shear strength from 1.829 MPa for neat samples to 2.186 MPa. The improvement in load-carrying capacity is evident from the larger area under the load–displacement curve (Fig. 7a), indicating that 1.0 wt.% graphene strengthens the SLJs. This enhancement increases resistance to shear forces and promotes higher energy absorption during loading. At this concentration, the adhesive effectively distributes stress across the joint interface under shear loading conditions.

In contrast, raising the graphene content to 2.0 wt.% results in a 51.56% decrease in shear strength, dropping it to 0.886 MPa, which is lower than the neat sample strength of 1.829 MPa (Fig. 7b). This reduction in performance can be attributed to weakened interfacial bonding at higher graphene concentrations, which reduces structural cohesion and limits the material’s ability to resist shear forces. The poor bonding observed at 2.0 wt.% likely leads to premature failure at lower loads, as the SLJ cannot sustain shear stresses without cracking at the adhesive interface. Consequently, this composition results in a less effective load transfer mechanism, compromising both load-carrying capacity and energy absorption.

Further examination of the shear strength trend clarifies the effect of graphene content. The short glass fibre-reinforced PLA-based joints exhibit an increase in shear strength with graphene contents of 0.5 wt.%, 1.0 wt.%, and 1.5 wt.%, achieving shear strength values of 2.139 MPa, 2.186 MPa, and 1.968 MPa, respectively. These values correspond to improvements of 16.49%, 19.51%, and 7.59% over the 1.829 MPa strength of the neat samples. This trend indicates an initial enhancement in shear resistance due to improved bonding, translating into higher load-bearing performance at moderate graphene concentrations. At a graphene concentration of 2.0 wt.%, the shear strength decreases to 0.886 MPa. This indicates a critical threshold where adding more graphene negatively affects bonding and limits effective stress distribution. The load–displacement curves for samples containing 2.0 wt.% graphene show reduced energy absorption and a lower peak load. Thus, it is concluded that the optimal graphene content is between 0.5 wt.% and 1.0 wt.%. At these lower concentrations, enhancements in bonding significantly improve both shear strength and load-carrying capacity without compromising structural integrity.

**Fig. 8:**
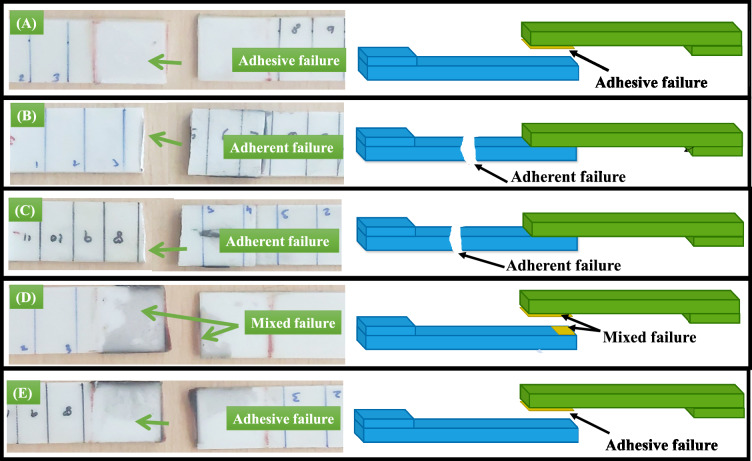
Failure surfaces of lap joints subjected to shear loading (**a**) Neat lap joint, (**b**) joint with 0.5 wt.% graphene, (**c**) joint with 1.0 wt.% graphene (**d**) joint with 1.5 wt.% graphene (**e**) joint with 2.0 wt.% of graphene.

Figure 8 displays the failure surfaces of the shear-tested adhesively bonded lap joints made from 3D-printed short glass fibre-reinforced PLA adherents, both reinforced with and without graphene. Examining these failure surfaces reveals critical insights into how graphene influences both adhesive and cohesive behaviours.

In the neat joints, failure occurred at the adhesive interface, indicating an adhesive failure (Fig. 8a). However, when graphene was added at concentrations of 0.5 wt.% and 1.0 wt.%, the failure mode changed significantly; these joints exhibited adherent failure (Fig. 8b, c). This shift suggests that graphene reinforcement enhances the adhesive strength to a level that surpasses the strength of the PLA-based adherends. Consequently, the substrate fails under loading instead of the adhesive interface. The mechanism behind this improvement likely arises from graphene’s ability to enhance interfacial adhesion through increased surface energy and mechanical interlocking, allowing for better load transfer across the adhesive joint. As a result, the strengthened bond between the adhesive and adherent enables the joint to endure higher shear forces before failure occurs within the fibre-reinforced PLA adherent rather than at the adhesive interface.

At 1.5 wt.% graphene addition, the failure surface shows a combination of cohesive and adhesive failures (Fig. 8d), indicating a transitional point in the composite’s joint’s mechanical response. At this concentration, graphene reinforcement strengthens the adhesive bond while also beginning to affect the internal cohesive integrity of the PLA-based composite. The presence of both cohesive and adhesive failures suggests that the bond strength and material strength are closely matched, leading to failures occurring partly within the substrate and partly at the adhesive boundary. This mixed failure mode implies that 1.5 wt.% graphene achieves a balance between adhesion and cohesion, enhancing shear strength without compromising the structural integrity of the composite joint.

When the graphene content rises to 2.0 wt.%, the failure mode predominantly reverts to adhesive failure (Fig. 8e), with the joint failing along the adhesive-adherent interface at a lower load than the neat sample. This reduction in performance is likely due to aggregation, where excess graphene forms clusters instead of dispersing uniformly within the adhesive. These clusters may weaken interfacial bonding by reducing the effective contact area and disrupting stress distribution across the joint, ultimately limiting load transfer efficiency. As a result, the adhesive is unable to withstand the applied shear forces, leading to premature adhesive failure with a lower load-bearing capacity than the neat joint. This trend emphasises the importance of controlled graphene content, as excessive graphene can reduce adhesive strength, highlighting an optimal range of 0.5–1.0 wt.% for maximising shear performance through improved adhesion.

**Fig. 9:**
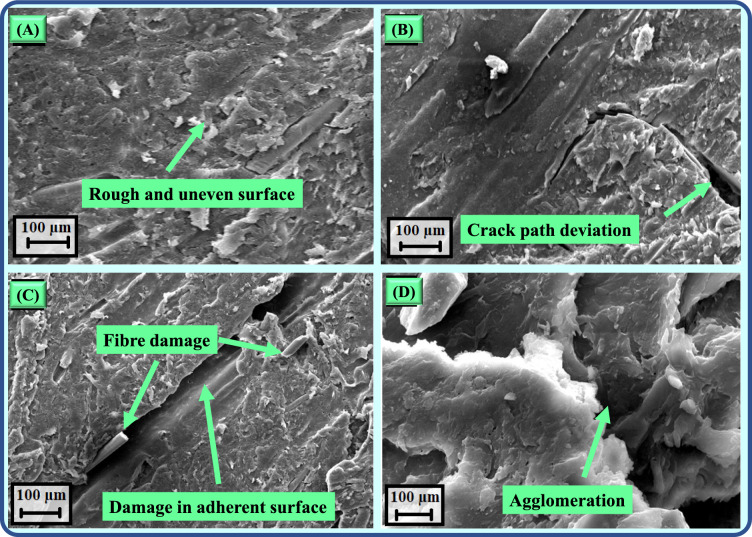
FE-SEM images of lap joints subjected to shear loading (**a**) joint with 0.5 wt.% graphene, (**b**) joint with 1.0 wt.% graphene, (**c**) joint with 1.0 wt. % graphene (**d**) joint with 2.0 wt.% graphene.

The SEM analysis shows that adding graphene significantly affects the microstructure and failure mechanisms of adhesively bonded lap joints made from 3D-printed short glass fibre-reinforced PLA adherents (Fig. 9). At a concentration of 0.5 wt.% graphene, the surface morphology becomes rougher and more uneven, which enhances adhesion at the interface (Fig. 9a). This improvement leads to better load transfer and increased energy absorption during shear loading. The enhanced adhesion is due to graphene’s high surface area, which promotes strong interfacial bonding and more effective stress distribution.

As the graphene content increases to 1.0 wt.%, a change in crack propagation patterns is observed. Graphene alters stress pathways, causing cracks to follow longer routes and thereby increasing the fracture toughness of the joint (Fig. 9b). Moreover, the rough surface morphology of graphene-reinforced joints adds friction at the fracture interface, contributing further to energy absorption and resistance to crack propagation under shear loads. At 1.0 wt.% graphene, damage to the adherent is seen, suggesting that the joint strength exceeds the strength of the adherent (Fig. 9c). This indicates a need for a stronger adhesive matrix to leverage the reinforcement capabilities of graphene fully. However, when the concentration reaches 2.0 wt.%, the graphene particles start to agglomerate (Fig. 9d). This accumulation creates localised stress points that reduce shear strength by disrupting uniform load transfer across the interface.

### Free vibrational analysis

**Fig. 10:**
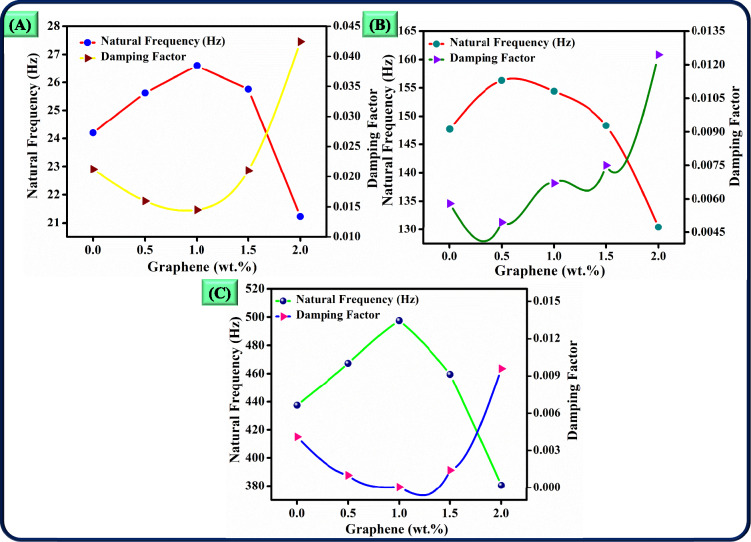
Free vibrational behaviour of adhesively bonded lap joints made from 3D-printed short glass fibre-reinforced PLA adherents (**a**) Mode 1 (**b**) Mode 2 (**c**) Mode 3.

The study on the vibrational response of adhesively bonded lap joints made from 3D-printed short glass fibre-reinforced PLA adherents demonstrates that the addition of graphene significantly affects both the natural frequencies and damping behaviour of the joints (Fig. 10a-c). This effect largely depends on the concentration of graphene in the adhesive matrix, as illustrated by the data from different vibrational modes.

For natural frequencies, an initial increase is observed with the addition of graphene up to 1.0 wt.%. At this concentration, graphene is well-dispersed within the epoxy matrix, resulting in a noticeable enhancement of stiffness. Graphene’s exceptionally high young’s modulus and tensile strength aid the PLA-based adhesive in improving load-bearing capability and facilitating more effective stress transfer across the joint. As stiffness increases, the natural frequencies of the structure also rise, as stiffer materials tend to vibrate at higher frequencies. This is evident in Mode 1, where the natural frequency increases from 24.202 Hz at 0 wt.% to 26.6 Hz at 1.0 wt.% graphene, indicating optimised stress transfer.

However, when the concentration exceeds 1.5 wt.%, the natural frequencies begin to decline. At this higher concentration, graphene particles are more likely to agglomerate within the epoxy matrix. This aggregation disrupts the structural uniformity and reduces the composite’s overall stiffness, as the clumped particles are less effective at reinforcing the material. Furthermore, the excess graphene lowers the natural frequencies. For instance, the natural frequency of Mode 1 decreases to 21.23 Hz at 2.0 wt.% graphene, indicating that the effects of aggregation offset the benefits of stiffness enhancement.

Damping behaviour exhibits a contrasting trend with increasing graphene content. At low levels of graphene (0.5–1.0 wt.%), the damping factor decreases, suggesting that well-dispersed graphene strengthens the composite without increasing internal friction or energy dissipation. In this range, the addition of graphene leads to a more resilient matrix that vibrates with lower energy loss; for example, the damping factor for Mode 1.0 reduces from 0.02122 at 0 wt.% to 0.0145 at 1.0 wt.% graphene. However, when the graphene content exceeds 1.0 wt.%, the damping factor starts to rise. Higher concentrations of graphene create more internal interfaces and contribute to accumulation, which increases frictional and interfacial energy dissipation during vibration.

The increase in the damping factor is particularly noticeable at 2.0 wt.% graphene, where the damping factor for Mode 1 reaches 0.04244, indicating significant energy dissipation. The presence of more filler-matrix interfaces and microstructural irregularities due to aggregated graphene particles promotes greater energy absorption, which can be beneficial for applications requiring high damping to reduce vibrational effects. Thus, while increased graphene content reduces stiffness and natural frequencies, it enhances the material’s damping capacity.

In conclusion, the optimal concentration of graphene depends on the specific application requirements. For applications that demand higher stiffness and natural frequencies, a lower concentration (up to 1.0 wt.%) of graphene is ideal, as it maximises stress transfer without increasing damping. Conversely, applications that benefit from vibration suppression and energy dissipation may find higher graphene concentrations (greater than 1.5 wt.%) advantageous due to the increased damping. This dual effect of graphene on natural frequencies and damping provides valuable insights for tailoring material properties in vibration-sensitive engineering applications**.**

### Prediction of failure loads through ANN

**Fig. 11:**
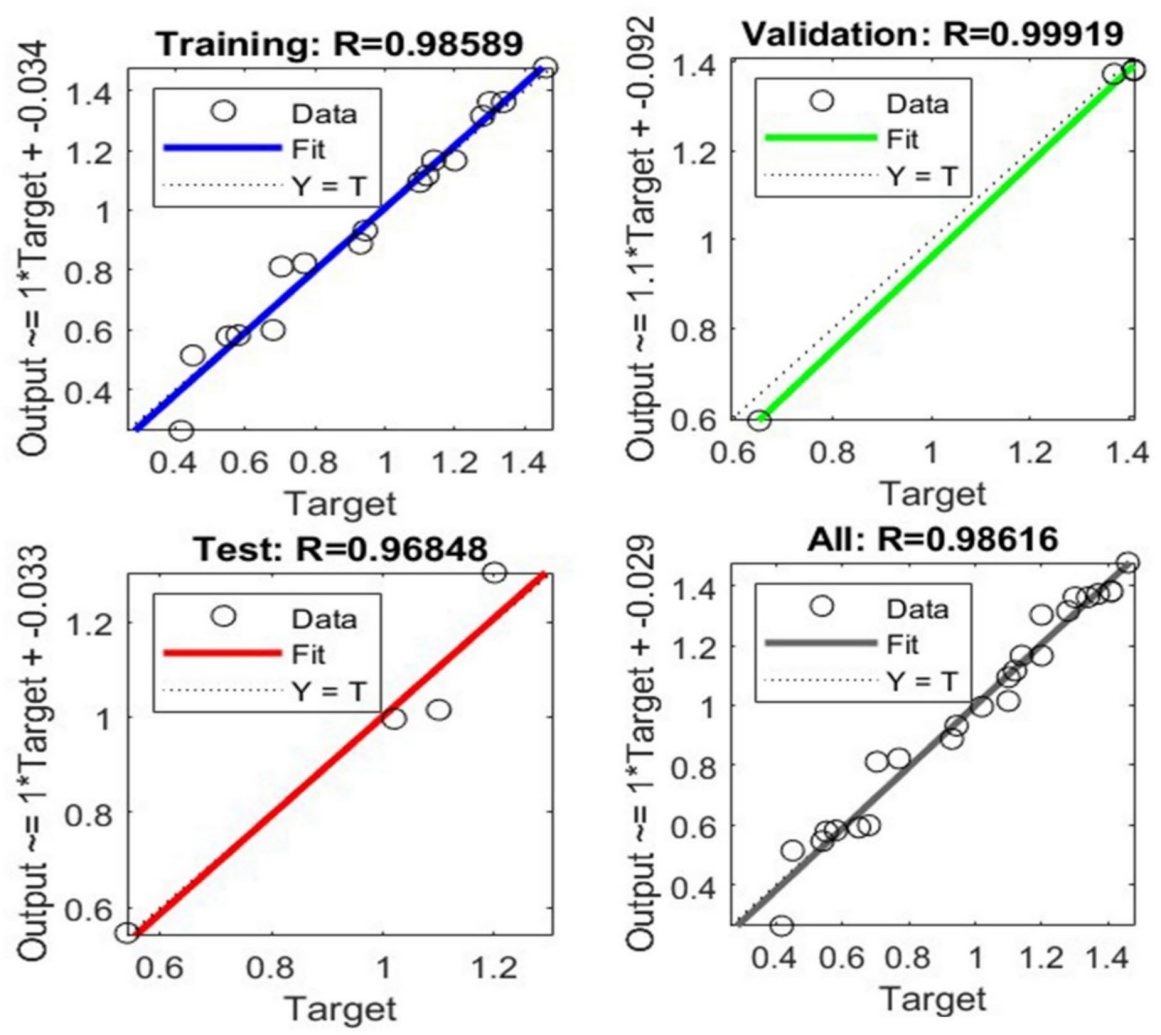
ANN regression plot showing training, validation, and testing results, along with an overall representation.

The present study aims to predict the failure load in adhesively bonded lap joints made from 3D-printed short glass fibre-reinforced PLA adherents using ANN and backpropagation techniques. These ANN were employed to manage the rate of errors that occurred during the testing and development phases. A correlation coefficient of 0 suggests a weak relationship, whereas a regression coefficient of 1 indicates a strong connection. To gain a deeper insight into the input and output variables, each training session consisted of 17 epochs. From the total of 25 datasets, 17 were utilised as training inputs for the network, while four were employed for validation purposes and another four for testing. The ANN generated a regression plot, which is displayed in Fig. 11, which illustrates the data for training, validation, testing and an overall representation. In the assessment of peak load prediction for testing the shear performance of lap joints, the correlation variables demonstrated strong performance across different datasets. The training set achieved an R^2^ value of 0.9859, while the testing set showed an exceptionally high R^2^ of 0.968. The validation set also performed well with an R^2^ of 0.99919. When considering all datasets together, the overall R^2^ value was calculated to be 0.9861, indicating a high level of predictive accuracy. The x-axis displays the experimental target values, while the vertical axis shows the load predictions generated by the trained ANN. The dashed line indicates the optimal correlation that can be established, while the solid, thicker lines show the actual correlations between the target and output values. The backpropagation algorithm modifies the weights to achieve an appropriate mean square error. The system’s error is calculated as the discrepancy between the output it produces and the desired target value provided to it. The optimal validation performance of the network is determined by the point at which the mean squared error converges for the training, validation and test datasets. The precise prediction of the failure load and peak load using ANN contributed significantly to efficiently obtaining remarkable results.

## Conclusions

This experimental study investigates the effect of graphene-integrated adhesive on the shear and vibrational response of adhesively bonded lap joints made from 3D-printed short glass fibre-reinforced PLA adherents. Based on the shear and vibrational analysis and observations, the following conclusions can be drawn.

- The tensile analysis indicated that the 0º oriented samples had a tensile strength of 30.7 MPa, demonstrating a 14.98% improvement over the 26.7 MPa observed in 45º samples and a 31.19% increase over the 23.4 MPa strength in 90º samples. For the 0º samples, the continuous fibre alignment provided resistance to crack propagation, enabling the material to endure higher deformation and absorb more energy.
- The short glass fibre-reinforced PLA-based joints showed improved shear strength when graphene was added at concentrations of 0.5 wt.%, 1.0 wt.%, and 1.5 wt.%, yielding values of 2.139 MPa, 2.186 MPa, and 1.968 MPa, respectively. These represented improvements of 16. 49%, 19. 51%, and 7.59% compared to the 1.829 MPa strength of the neat samples. In contrast, increasing the graphene content to 2.0 wt.% resulted in a 51.56% decrease in shear strength, reducing it to 0.886 MPa, which is lower than the strength of the neat adhesive, indicating that excessive graphene addition can lead to performance degradation due to particle aggregation.
- The failure analysis revealed that at 0.5 wt.% and 1.0 wt.% graphene, the failure mode transitioned from adhesive to adherent, suggesting improved bonding at the interface. This transition is allied to the enhanced load transfer enabled by graphene at optimal concentrations. The FESEM analysis revealed that as the graphene content increases to 1.0 wt.%, changes in crack propagation patterns occur. Graphene modifies the stress pathways, causing cracks to take longer routes, which in turn enhances the fracture toughness of the joint.
- Modal analysis showed that graphene concentrations up to 1.0 wt. % increased stiffness and natural frequencies, suggesting improved dynamic performance of the joints. However, higher concentrations (above 1.5 wt.%) led to an increase in damping capacity, contributing to vibration suppression rather than stiffness enhancement.
- The assessment of peak load predictions using data-driven approaches showed high accuracy, with R^2^ values of 0.9859 for the training set, 0.968 for the testing set, and 0.99919 for the validation set. This reflects a strong correlation and reliable predictive capability. Overall, the findings confirm that graphene integration within 0.5 wt.% to 1.0 wt.% is effective in enhancing both shear and vibrational properties of adhesively bonded lap joints made from 3D-printed short glass fibre-reinforced PLA adherent joints.

## Data Availability

The datasets used and/or analysed during the current study are available from the corresponding author upon reasonable request.
